# Novel Use of N-Acetylcysteine in Management of Tyrosine Kinase Inhibitor Induced Acute Liver Injury

**DOI:** 10.7759/cureus.6251

**Published:** 2019-11-27

**Authors:** Tarang Patel, Tushar Tarun, Dania Hudhud, Armin Krvavac

**Affiliations:** 1 Internal Medicine, University of Missouri Healthcare, Columbia, USA; 2 Cardiology, University of Missouri School of Medicine, Columbia, USA; 3 Internal Medicine, University of Missouri School of Medicine, Columbia, USA; 4 Pulmonary & Critical Care, University of Missouri Healthcare, Columbia, USA

**Keywords:** pazopanib, n-acetylcysteine, acute liver failure, acute liver injury, transaminitis, non-acetaminophen induced acute liver failure, critical care, tyrosine kinase inhibitors (tkis)

## Abstract

Tyrosine kinase inhibitors (TKIs) have been adopted in the treatment of a variety of malignancies. Despite their popularity, the underlying mechanism of the adverse effects seen with the use of TKIs is not completely understood. Acute liver injury is a known side effect of many of these drugs. Some papers have demonstrated that N-acetylcysteine may have a role in non-acetaminophen induced acute liver failure (NAI-ALF). There is little evidence supporting the use of N-acetylcysteine in the treatment of tyrosine kinase inhibitor-induced acute liver injury. This case report adds to the limited body of existing knowledge.

We present a 67-year-old Caucasian female with a past medical history of anxiety, hyperlipidemia, in utero exposure to diethylstilbestrol (DES), and well-differentiated angiosarcoma of the right breast. She achieved remission for approximately six years after mastectomy with adjuvant chemotherapy and radiation. Subsequent surveillance imaging revealed new hepatic and cervical lesions. Further investigation with cutaneous biopsy near the occipital region confirmed recurrent metastatic angiosarcoma. The patient was started on high-dose pazopanib and initially tolerated the TKI without any adverse effects. However, after approximately two weeks of therapy, she began to experience dark colored urine, myalgias, and fatigue. These symptoms, along with significant elevations in liver enzymes (alanine transaminase of 1377 units/L, aspartate transaminase of 1212 units/L), prompted admission for evaluation of acute liver injury. The etiology of the acute liver injury was suspected to be secondary to TKI therapy. Treatment with intravenous N-acetylcysteine was initiated for non-acetaminophen induced acute liver failure (NAI-ALF) and resulted in a dramatic improvement in transaminases before discharge.

Evidence suggests that there is a beneficial role for N-acetylcysteine in the management of NAI-ALF. However, when it comes specifically to the management of TKI induced acute liver injury, there is limited evidence to support its use. This case report highlights a possible use of N-acetylcysteine in the management of TKI mediated acute liver injury. Additional studies should be conducted to determine the role N-acetylcysteine plays in the management of TKI mediated liver injury.

## Introduction

The use of N-acetylcysteine (NAC) in the setting of acetaminophen-induced acute liver failure (AI-ALF) has been well studied and has become the standard of care in the management of this condition. There has been limited research regarding the use of NAC in the management of non-acetaminophen induced acute liver failure (NAI-ALF). NAC may produce an anti-inflammatory and antioxidant effect in the setting of NAI-ALF [1]. Additionally, NAC is thought to improve oxygenation via vasodilation of microcirculatory blood flow to vital organs [2]. Furthermore, the use of NAC in NAI-ALF has demonstrated a statistically significant mortality benefit and an association with a shorter length of hospitalization [3].

The use of tyrosine kinase inhibitors (TKI) in the management of malignancy has increased dramatically in the last decade. The underlying mechanism of the adverse effects seen with this class of novel drugs is still under investigation; however, acute liver injury has been reported with several TKIs [4-6]. In the appropriate clinical setting, TKI induced acute liver injury should be included in the differential diagnosis when considering possible etiologies of NAI-ALF. This case report discusses the novel use of NAC in the management of TKI induced acute liver injury. 

## Case presentation

**Figure 1:**
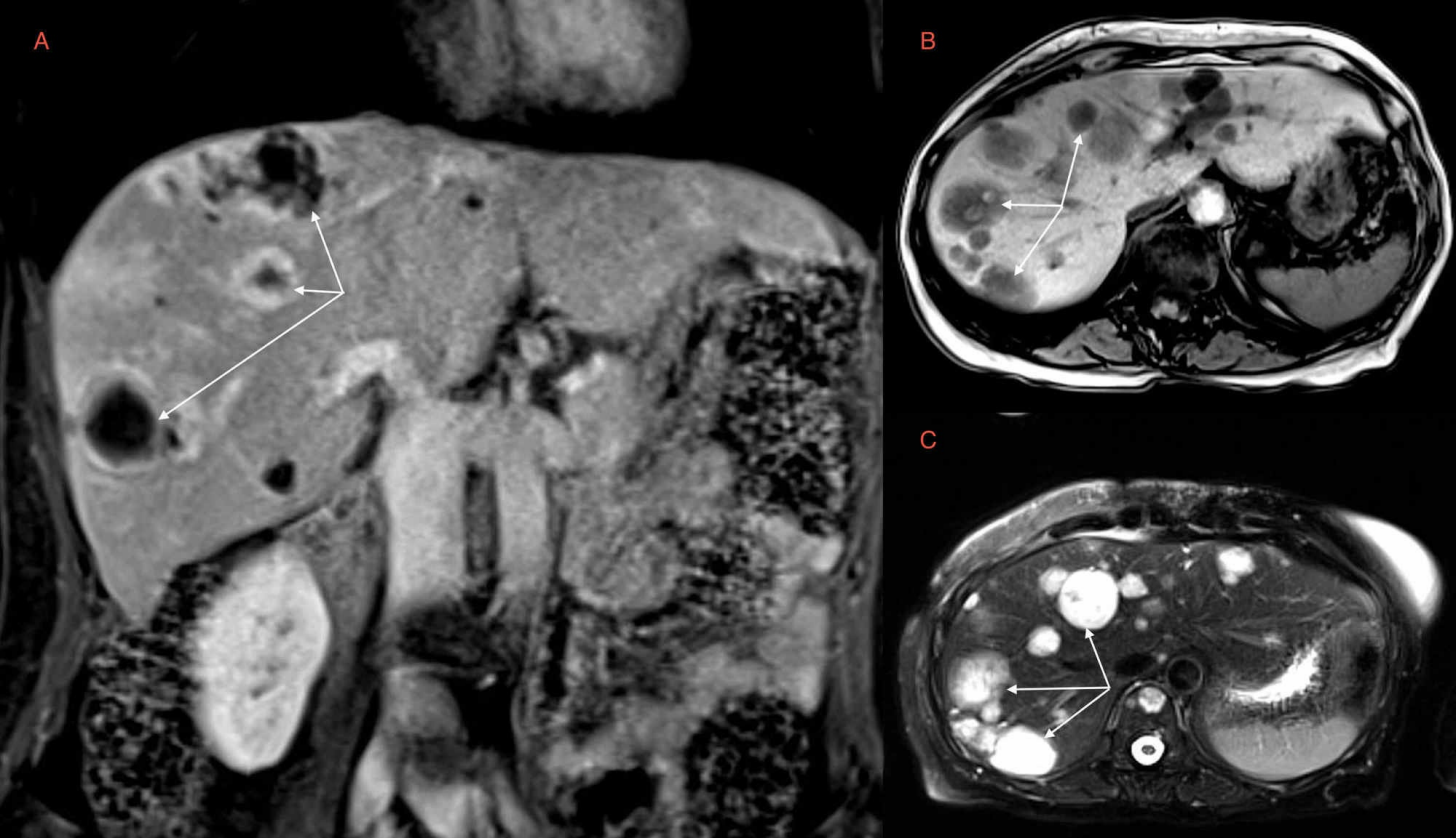
Abdominal Magnetic Resonance Imaging (A) T1 weighted coronal section, (B) T1 weighted axial section, and (C) T2 weighted axial section of abdominal magnetic resonance imaging with arrows highlighting multiple enhancing lesions that were concerning for recurrence of malignancy

The patient is a 67-year-old Caucasian female with a past medical history of anxiety, hyperlipidemia, in utero exposure to diethylstilbestrol (DES), and well-differentiated angiosarcoma of the right breast that was initially diagnosed via a core biopsy in 2011. She underwent right mastectomy with adjuvant radiotherapy and chemotherapy (gemcitabine-Taxotere). She achieved remission of her disease for approximately six years without any evidence of malignancy. However, subsequent surveillance computed tomography of the abdomen in 2017 revealed new scattered sub-centimeter enhancing hepatic lesions. Further evaluation of these hepatic lesions with magnetic resonance imaging (MRI) redemonstrated multiple sub-centimeter enhancing liver lesions (Figure 1) and a soft tissue mass in the cervical region, which was highly concerning for metastatic angiosarcoma. Ultimately, the patient underwent tissue biopsy, which confirmed metastatic angiosarcoma. She was subsequently enrolled in a clinical trial with high-dose pazopanib (NCT01462630).

The patient initially tolerated the TKI therapy (pazopanib) without any adverse effects. However, after approximately two weeks of therapy, she began to experience dark colored urine, myalgias, and fatigue. Subsequent evaluation at a local urgent care center revealed a new elevation in liver enzymes with alanine transaminase (ALT) of 522 units/L and aspartate transaminase (AST) of 456 units/L. These laboratory abnormalities prompted a referral to a tertiary care center for further evaluation, where repeat laboratory tests showed a significant increase in liver enzymes (ALT 1377 units/L and AST 1212 units/L). Additional testing revealed an international normalized ratio of 1.1, prothrombin time of 12.7 seconds, alkaline phosphatase 275 units/L, bilirubin of 1.5 mg/dL, and albumin of 4.3 g/dL. Serum acetaminophen levels were undetectable. An abdominal ultrasound of the right upper quadrant demonstrated unremarkable liver size, appearance, and echogenicity without intrahepatic ductal dilation or masses. Additionally, vascular ultrasound of the abdomen revealed no filling defects in the hepatic vein or main portal vein. Further studies, including serologies for herpes simplex virus, Ebstein Barr virus, cytomegalovirus, viral hepatitis, anti-nuclear antibody, and anti-smooth muscle antibody, were negative. Evaluation ultimately revealed no anatomic, infectious, or autoimmune etiologies for the patient's acute liver injury. The etiology of acute liver injury was, therefore, suspected to be secondary to the use of TKI (pazopanib).

**Figure 2:**
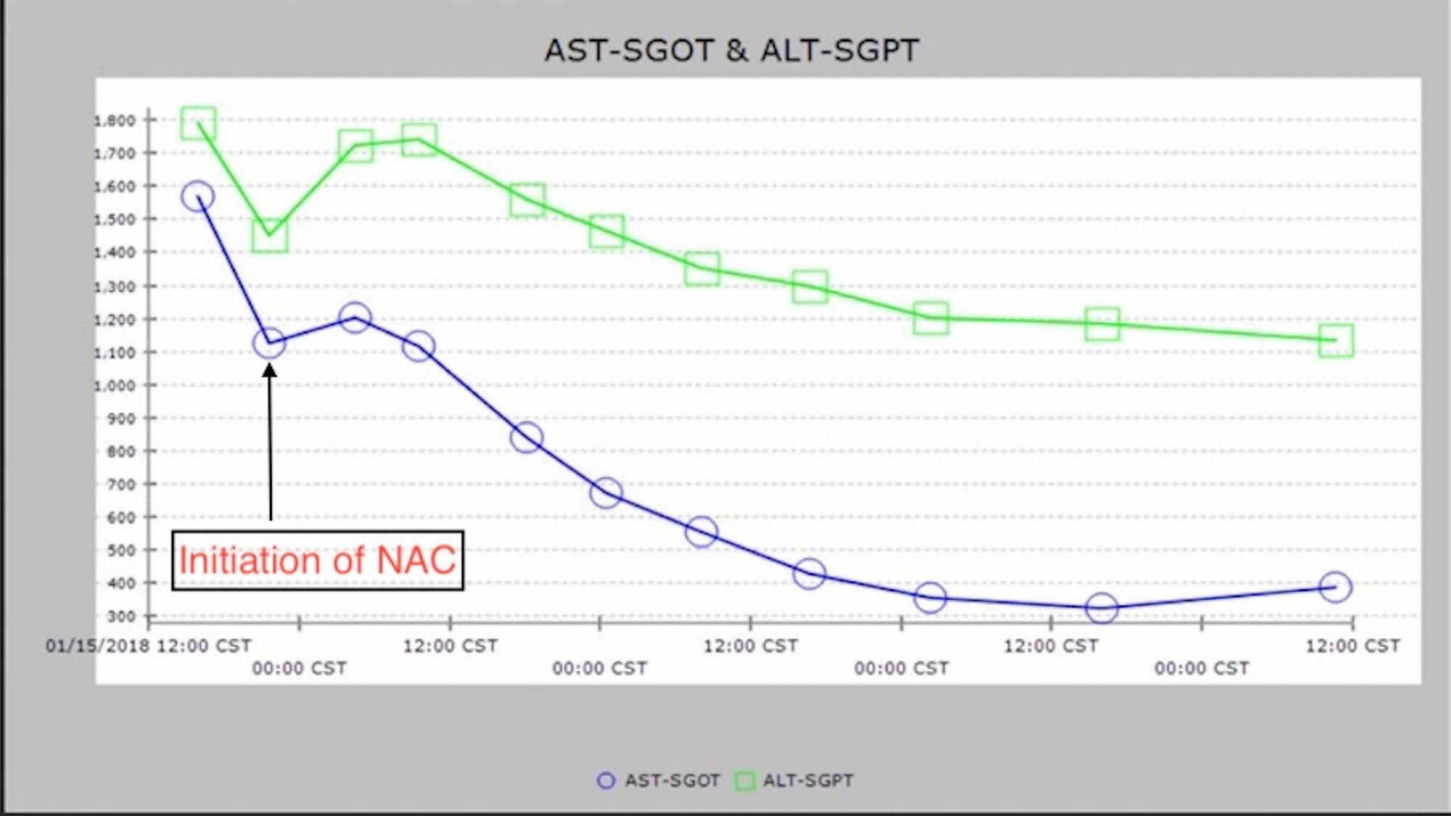
Trend of Liver Enzymes During Hospitalization AST-SGOT: aspartate aminotransferase, ALT-SGPT: alanine aminotransferase, NAC: N-acetylcysteine

Prompt treatment with intravenous NAC for NAI-ALF was initiated on admission, and pazopanib was discontinued. The patient was monitored with serial complete metabolic panels and coagulation studies every eight hours. The acute liver injury rapidly improved during the hospital course, and the patient was ultimately discharged home 48 hours post-admission (Figure 2). 

Close follow-up was arranged with the hepatology clinic, where repeat laboratory testing showed continued improvement in liver enzymes.

## Discussion

A limited body of knowledge describes the use of NAC in the setting of NAI-ALF. The available literature is even more limited concerning the use of NAC in the context of TKI induced acute liver injury.

One of the early mentions of the use of NAC in NAI-ALF was described in a prospective study of 91 patients studied over a seven-year period. In this study, the primary causes of NAI-ALF were from viral hepatitis (hepatitis E, hepatitis B, and hepatitis D) as well as drug-induced liver injury from tuberculosis drug therapy. However, none of the studied patients demonstrated NAI-ALF from TKI. A drawback of this study was a historical control group derived from chart review. Despite these limitations, the study showed that the use of NAC for NAI-ALF in a non-liver transplant center is a relatively safe intervention that significantly reduced mortality and length of hospitalization [3]. 

The use of NAC in NAI-ALF is further supported by a prospective randomized case-control trial, conducted over two years, which assessed the effect of NAC therapy on mortality in patients with NAI-ALF. The trial enrolled 80 patients that were randomized to NAC versus placebo. Once again, the majority of the causes of NAI-ALF were secondary to acute viral hepatitis and tuberculosis drug therapy. There were no cases of NAI-ALF due to TKI therapy. Nevertheless, significant improvement in length of hospitalization and mortality with NAC was once again demonstrated, with reported 28% mortality in the NAC group compared to 53% mortality in the placebo group [7].

The overall evidence points to a decrease in mortality and hospital length of stay when NAC is used in the treatment of NAI-ALF [3,7]. However, this evidence is limited to two single-center trials. Furthermore, neither trial included or addressed the use of NAC in TKI induced NAI-ALF. In this particular case, it is difficult to ascertain whether improvement in the patient’s acute liver injury was a result of the cessation of TKI (pazopanib) or the initiation of NAC. Considering NAC has an excellent safety profile with minimal risk of side-effects, it is not unreasonable to treat patients with NAC for TKI induced NAI-ALF given the potential benefit seen in other NAI-ALF [8]. Further case reports and prospective studies will need to be conducted to determine if there is a significant effect on mortality and length of hospitalization in TKI induced NAI-ALF.

## Conclusions

There is evidence that exists to suggest that there is a role for N-acetylcysteine in the management of non-acetaminophen induced acute liver failure. However, when it comes specifically to the management of tyrosine kinase inhibitor-induced acute liver injury, there is no large body of evidence to support its use. This case report highlights the possible use of NAC in the management of TKI mediated acute liver injury. Further case reports, case series, and prospective trials are needed to build the body of evidence in favor of this particular intervention.
